# Elevated Serum Total Bilirubin Concentrations Are Negatively Associated with Diabetic Retinopathy among the Chinese Northeastern Population

**DOI:** 10.1155/2018/6539385

**Published:** 2018-03-27

**Authors:** Dan Zhang, Wei Zhang, Shi Jin, Wei Wang, Dan Guo, Lu Wang

**Affiliations:** Department of Endocrinology, Fourth Hospital of China Medical University, Shenyang 110032, China

## Abstract

**Objective:**

To evaluate the association between serum total bilirubin concentration (STBC) and diabetic retinopathy (DR) among the Chinese northeastern population.

**Methods:**

A cross-sectional study was conducted in Liaoning between January 2015 and May 2017.

**Results:**

A total of 742 subjects (419 men and 323 women) with type 2 diabetes mellitus (DM) who visited an ophthalmic clinic were included in this study. The mean age of the subjects was 59.55 ± 10.63 years, and 43.5% of the subjects were women. The mean of DM duration was 11.01 ± 7.35 years. STBC were negatively correlated with DM duration, urea nitrogen, serum creatinine, uric acid, and urine microalbumin. After adjusting for confounding factors, as a continuous variable, STBC was inversely associated with the risk of DR in total subjects (OR: 0.95, 95% CI: 0.93–0.99). When STBC was used as a tertiary variable, compared with the first tertile, the OR in the third tertile was 0.37 (95% CI: 0.22–0.64) in total subjects.

**Conclusion:**

Our results demonstrate that a significant negative association was found between STBC and DR. STBC might be an early clinical marker for predicting the occurrence of DR.

## 1. Introduction

As an important noncommunicable disease, diabetes mellitus (DM) has become one of the major public health problems in the world. DM does not only have an important influence on physical health and the quality of life but can also bring about multiple complications and negatively affect a person's mental health and well-being [1]. Currently, due to population ageing, urbanization, and lifestyle changes, the number of people with DM has increased sharply all over the world [2]. The International Diabetes Federation (IDF) reported that there were about 382 million diabetic patients in 2013, and almost 600 million people would develop DM by 2035 [3]. In America, there were 29.1 million diabetic patients in 2014, and it is predicted that about 50% of all Americans will develop prediabetes or diabetes by 2020 [4]. By 2035, Asia will be the center of the DM epidemic; the four Asian countries (China, India, Indonesia, and Japan) might have the largest diabetes populations in the world [5].

Bilirubin is one of the end products of heme catabolism and has been shown to have antioxidant and anti-inflammatory effects [6]. Due to the effects of heme oxygenase (HO), which contain two forms: HO-1 and HO-2, cyclic tetrapyrrole heme is divided into biliverdin, carbon monoxide (CO), and ferrous iron (Fe^2+^). Bilirubin concentration increases as higher HO-1 expression increases [7]. It has been reported that the HO-1 system can act protectively in a variety of models of diseases, which included diabetic retinopathy (DR) [8]. Due to the potential role of oxidative stress in the pathogenesis of DR and the antioxidant and cytoprotective effects of bilirubin, the number of studies on the association between serum total bilirubin concentration (STBC) and the risk of DR was increasing, some studies found that elevated STBC might have a protective effect on DR [9, 10], and others did not find similar results [11]. In the meantime, some studies reported that serum bilirubin concentration was not only affected by age, gender, and BMI but also affected by altitude and ethnicity [12]. All these factors could influence the biological effects of bilirubin on the human body. China has a vast territory; there are great differences in demographic characteristics and living and eating habits between the northern and southern population [13], but the number of high-quality studies on the association between STBC and risk of DR was too small. Therefore, we first performed a study on the northeastern Chinese population to analyze the association between STBC and risk of DR.

## 2. Materials and Methods

### 2.1. Participants and Study Design

All subjects with type 2 DM who visited the ophthalmic clinic in the Fourth Hospital of China Medical University between January 2015 and May 2017 were retrospectively included in the study. Type 2 DM patients were defined as self-reported doctor-diagnosed diabetes or taking antidiabetic medications or fasting glucose concentration higher than or equal to 7.0 mmol/L according to the World Health Organization (WHO) criteria [14]. DR was defined by an ophthalmologist following an eye examination. Fundus photographs taken for both eyes were analyzed and graded to confirm the presence and severity of DR. DR mainly included nonproliferative and proliferative types. Nonproliferative DR showed one or more of the following symptoms: microaneurysm, hemorrhage, exudates, or microvascular abnormalities; proliferative DR showed the generation of new vessels and fibrosis [15].

We excluded (1) patients who had type 1 DM (defined as presentation with diabetic ketoacidosis, acute hyperglycemia symptoms with heavy ketonuria, or the continuous requirement of insulin in the year succeeding diagnosis); (2) patients who had hepatobiliary, hematological system, cardiopulmonary dysfunction, cancer, and other severe chronic diseases; (3) patients who also had other fundus lesions; and patients (4) when the doctor could not see the fundus due to refractive medium opacity.

This study was approved by the ethical committee of the Fourth Hospital of China Medical University (EC-2015-KS-030). Due to the retrospective nature of this study, the harm to the participants is not more than the minimal risk; the ethical committee of the Fourth Hospital of China Medical University waived the requirement to obtain informed consent.

### 2.2. Collection of Demographic, Medical, and Laboratory Data

For this hospital-based cross-sectional study, basic demographic data from all subjects were collected from medical records, including sex, age, height, weight, duration of diabetes, systolic blood pressure (SBP), and diastolic blood pressure (DBP). The body mass index (BMI) was calculated as the ratio of weight in kilograms divided by the square of height in meters. Blood and urine samples taken on admission were used for the analysis during the study period. Laboratory data was measured from fasting blood samples using an autoanalyzer according to the manufacturer's instructions. Total cholesterol (TC), triglyceride (TG), high-density lipoprotein cholesterol (HDL-C), low-density lipoprotein cholesterol (LDL-C), urea nitrogen (BUN), serum creatinine (Cr), uric acid (UA), fasting plasma glucose (FPG), serum total bilirubin concentration (STBC), and urine microalbumin were measured on an ADVIA 2400 automatic biochemical analyzer (SIEMENS, Germany). Fasting insulin and C-peptide were measured on an ADVIA Centaur XP immunoassay analysis system (SIEMENS, Germany). Hemoglobin A1c (HbA1c) was measured on a TOSOH HLC-723G7 analyzer (Sysmex, Japan). Fibrinogen was measured on an ACL TOP700 automatic blood coagulation analyzer (Beckman, USA).

### 2.3. Statistical Analysis

Data were entered (double entry) using Microsoft Excel 2013. Statistical analysis was performed using SPSS 22.0 for Windows (SPSS Inc., Chicago, USA). Continuous variables were expressed as the means and standard deviations. Categorical variables were expressed by numbers and percentages. The normality test was used to check the distribution of continuous variables. *t*-test and one-way analysis of variance (ANOVA) were used for the comparison of continuous variables, and the chi-square test was used for the comparison of categorized variables. The correlation coefficient was determined using Pearson's coefficient to determine the association between STBC and various clinical parameters. Finally, multivariable logistic regression was performed to estimate the odds ratio (OR) and 95% confidence intervals (CIs) for DR according to STBC. Reported probabilities were two-sided; a difference was considered significant if the *P* value was less than 0.05.

## 3. Results

### 3.1. Clinical Characteristics of the Subjects

The clinical characteristics are shown in Table 1. The mean age of the subjects was 59.55 ± 10.63 years, and 56.5% of the participants were men. The mean of the DM duration was 11.01 ± 7.35 years. The subjects without DR had a shorter DM duration and lower SBP, DBP, TC, TG, LDL-C, BUN, Cr, UA, urine microalbumin, and fibrinogen. The STBC was significantly higher in the subjects without DR. There was no difference in age, BMI, FPG, HbA1c, HDL-C, Fasting insulin, and C-peptide.

### 3.2. Correlations between STBC and Various Clinical Parameters

In total subjects, STBC was negatively correlated with DM duration, SBP, TC, TG, LDL-C, BUN, Cr, UA, urine microalbumin, and fibrinogen, and there was no correlation with other related clinical factors. The detailed results on correlations between STBC and various parameters are shown in Table 2.

### 3.3. Comparison of Related Clinical Parameters in Each Tertile Group of STBC

The clinical characteristics of the subjects in each tertile group are shown in Table 3. In total subjects, DM duration, SBP, TC, TG, LDL-C, BUN, Cr, UA, fasting insulin, urine microalbumin, and fibrinogen in the third tertile were lower than those in the first tertile; SBP, TC, BUN, Cr, urine microalbumin, and fibrinogen in the second tertile were lower than those in the first tertile; and DM duration and UA in the third tertile was lower than those in the second tertile. By chi-square test, the percentage of DR in the third tertile was lower than that in the first tertile.

### 3.4. Association between STBC and the Risk of DR

In a multivariate analysis adjusted for sex, age, BMI, DM duration, SBP, DBP, FPG, HbA1c, TC, TG, HDL-C, LDL-C, BUN, Cr, UA, fasting insulin, urine microalbumin, C-peptide, and fibrinogen (Table 4; model 3), as continuous variables, STBC was inversely associated with the risk of DR (OR: 0.95, 95% CI: 0.93–0.99). When STBC was used as the tertiary variable, compared with the first tertile, the OR in the third tertile was 0.37 (95% CI: 0.22–0.64). The detailed results on the association between STBC and the risk of DR are shown in Table 4.

## 4. Discussion

In this study, we first analyzed the association between STBC and the risk of DR in the Chinese northeastern population. STBC was negatively correlated with DM duration, SBP, TC, TG, LDL-C, BUN, Cr, UA, urine microalbumin, and fibrinogen. Furthermore, we found that diabetic patients in the highest tertile of STBC have significantly decreased ORs for the risk of DR, even after adjusted for potential confounding factors in total subjects. We also found that STBC by one standard deviation was negatively associated with the risk of DR.

In recent years, DM has become a rapidly growing threat worldwide; it causes a huge burden for people affected by it and also has an important influence on physical health and life quality according to its multiple complications [1]. Thus, identifying high-risk diabetic individuals with a higher risk of complications is very important and may lead to improvements in preventing and decreasing the burden of this disease. Thus, in clinical practice, it is very important to find a simple and convenient indicator to predict the occurrence of DR. Due to its antioxidant and anti-inflammatory effects, the research field on bilirubin is one of the research hotspots in the world. The Heinz Nixdorf Recall study found that bilirubin showed a potential protective role in the atherosclerosis process [16]. Several studies have found that STBC was negatively associated with diabetic microvascular and macrovascular complications [17–19], and in 2017, a meta-analysis found that there was a negative association between STBC and diabetic complications [20]. A total of 674 patients with type 2 diabetes in Japan revealed that STBC was significantly lower in patients with retinopathy than in those without [10]. In 2014, Najam et al. found that diabetic patients in the higher STBC group were less likely (OR: 0.55; 95% CI: 0.33–0.91) to suffer from DR than patients in the lower STBC group in Shanghai [18]. Compared to domestic and international research results, our results were similar.

The biological mechanisms underlying the association between serum bilirubin levels and DR remain unclear, but there are several possible explanations. Oxidative stress is an important risk factor in the occurrence of DR [21]. As the end product of heme catabolism, bilirubin has a powerful antioxidant capacity. In the total antioxidant capacity of blood plasma, bilirubin was a major contributor; only 10 nM bilirubin scavenges a 10,000-fold higher concentration of hydrogen peroxide [22]. Bilirubin also plays an important role in physiological antioxidant by inhibiting the formation of reactive oxygen species through NADPH oxidase in the endothelial cell [23]. Fu et al. found that due to a lack in the enzyme uridine-diphosphate glucuronosyltransferase (UGT1A) and exhibiting an elevation of plasma bilirubin concentration, the mice reduced streptozotocin-induced pancreatic *β*-cell dysfunction by weakening oxidative stress [24]. Similar results were found in the studies on genetic variants. Abbasi et al. performed Mendelian randomization in a prospective cohort of 3381 participants, used rs6742078 located in UGT1A 1, and found that elevated STBC is causally associated with the risk of DM [25]. Several studies also found that low-grade inflammation plays a critical role in the development of diabetic retinopathy [16, 26]. Bilirubin also has anti-inflammatory properties through interfering with the expression of cell adhesion molecules, complement activity, and T cell differentiation [22]. Furthermore, clinical studies have demonstrated a negative relationship between serum bilirubin and C-reactive protein levels, which is a robust marker of inflammatory status [27]. In subjects with Gilbert syndrome, Tapan et al. found that there was a negative association between serum bilirubin concentration and soluble forms of CD40 ligand and P-selectin [28]. The modulatory effects of bilirubin on T regulatory cell differentiation were recently reported [29], further underlining the protective role of bilirubin in the pathogenesis of chronic inflammatory as well as in autoimmune conditions.

Our study had several limitations. First, due to the hospital-based cross-sectional study, the subjects were mainly collected from the ophthalmic clinic; therefore, there may be selection bias in the collection [30], and we could not directly infer the causal relationship between serum bilirubin concentration and the risk of DR. Second, serum bilirubin concentration was measure only once and, therefore, could not reflect the fluctuation and mean level. Taking these things into consideration, the results of our study may not be applicable to the general population or patients with type 2 diabetes. So then, our study aimed at revealing and exploring the relationship between serum bilirubin concentration and the risk of DR. In the future, several more prospective studies are needed to confirm the present findings in the Chinese population.

## 5. Conclusions

Currently, the measuring method of STBC in most hospitals is performed routinely and is not expensive to patients. Thus, STBC can be used easily by clinicians as one of the risk factors for the development of DR. Our results indicated that STBC could be considered a biomarker to predict the risk of DR.

## Figures and Tables

**Table 1:** Clinical characteristics of the subjects.

Characteristics	Total	Without DR	With DR	*P*
*n*	742	209	533	
Men (%)	56.5%	56.9%	56.3%	0.872
Age (Year)	59.55 ± 10.63	59.23 ± 10.90	59.67 ± 10.53	0.616
DM duration (year)	11.01 ± 7.35	7.03 ± 6.47	12.57 ± 7.09	<0.001
BMI (kg/m^2^)	25.01 ± 2.85	24.90 ± 3.37	25.06 ± 2.61	0.506
SBP (mmHg)	140.49 ± 20.23	137.91 ± 21.15	141.49 ± 19.78	0.03
DBP (mmHg)	83.62 ± 11.32	82.31 ± 12.11	84.14 ± 10.97	0.048
FPG (mmol/L)	9.68 ± 3.68	9.75 ± 3.89	9.65 ± 3.60	0.743
HbA1c (%)	9.30 ± 1.52	9.31 ± 1.90	9.30 ± 1.34	0.972
TC (mmol/L)	4.86 ± 1.05	4.52 ± 1.05	5.00 ± 1.02	<0.001
TG (mmol/L)	2.04 ± 1.77	1.64 ± 1.23	2.20 ± 1.92	<0.001
HDL-C (mmol/L)	1.15 ± 0.32	1.16 ± 0.29	1.14 ± 0.33	0.568
LDL-C (mmol/L)	2.99 ± 0.84	2.81 ± 0.88	3.06 ± 0.81	<0.001
BUN (mmol/L)	6.69 ± 3.83	5.25 ± 1.47	7.26 ± 4.29	<0.001
Cr (*μ*mol/L)	119.55 ± 127.32	87.28 ± 19.34	132.20 ± 147.86	<0.001
UA (*μ*mol/L)	310.86 ± 88.27	280.88 ± 73.57	322.61 ± 90.80	<0.001
Fasting insulin (*μ*U/mL)	13.35 ± 11.30	13.44 ± 11.66	11.32 ± 11.17	0.895
Urine microalbumin (mg/L)	70.88 ± 69.12	36.74 ± 54.42	84.27 ± 69.71	<0.001
C-peptide (ng/mL)	2.46 ± 1.20	2.50 ± 1.33	2.45 ± 1.14	0.647
Fibrinogen (g/L)	3.38 ± 1.22	3.15 ± 0.53	3.46 ± 1.39	0.002
STBC (*μ*mol/L)	13.95 ± 7.08	17.04 ± 8.98	12.75 ± 5.75	<0.001

**Table 2:** Correlation of STBC and various clinical parameters in total subjects.

Total		
Characteristics	*r*	*P*
Age (year)	0.018	0.620
DM duration (year)	−0.213	<0.001
BMI (kg/m^2^)	−0.065	0.079
SBP (mmHg)	−0.079	0.030
DBP (mmHg)	−0.006	0.880
FPG (mmol/L)	0.037	0.319
HbA1c (%)	0.029	0.424
TC (mmol/L)	−0.113	0.002
TG (mmol/L)	−0.118	0.001
HDL-C (mmol/L)	0.045	0.221
LDL-C (mmol/L)	−0.066	0.073
BUN (mmol/L)	−0.230	<0.001
Cr (*μ*mol/L)	−0.173	<0.001
UA (*μ*mol/L)	−0.149	<0.001
Fasting insulin (*μ*U/mL)	−0.077	0.035
Urine microalbumin (mg/L)	−0.176	<0.001
C-peptide (ng/mL)	−0.068	0.066
Fibrinogen (g/L)	−0.163	<0.001

**Table 3:** Comparison of clinical characteristics according to STBC tertiles in total subjects.

Characteristics	Q1	Q2	Q3
	(<10.81)	(10.81–14.61)	(>14.61)
*n*	248	250	244
Age (year)	58.92 ± 11.02	60.02 ± 10.51	59.70 ± 10.35
DM duration (year)	12.12 ± 7.47	12.08 ± 7.32	8.79 ± 6.77^ab^
BMI (kg/m^2^)	25.03 ± 2.88	25.16 ± 2.90	24.84 ± 2.75
SBP (mmHg)	144.19 ± 21.38	138.39 ± 19.02^a^	138.87 ± 19.77^a^
DBP (mmHg)	83.61 ± 11.10	82.71 ± 11.68	84.57 ± 11.14
FPG (mmol/L)	9.60 ± 4.19	9.58 ± 3.48	9.87 ± 3.32
HbA1c (%)	9.25 ± 1.43	9.38 ± 1.52	9.28 ± 1.62
TC (mmol/L)	5.02 ± 1.14	4.80 ± 0.97^a^	4.77 ± 1.02^a^
TG (mmol/L)	2.28 ± 1.95	1.98 ± 1.58	1.85 ± 1.74^a^
HDL-C (mmol/L)	1.12 ± 0.34	1.15 ± 0.32	1.17 ± 0.30
LDL-C (mmol/L)	3.07 ± 0.86	2.96 ± 0.81	2.93 ± 0.83
BUN (mmol/L)	8.14 ± 5.18	6.41 ± 3.30^a^	5.51 ± 1.64^ab^
Cr (*μ*mol/L)	157.88 ± 188.64	109.82 ± 100.88^a^	90.55 ± 19.60^a^
UA (*μ*mol/L)	327.97 ± 96.72	311.42 ± 89.86^a^	292.90 ± 73.17^ab^
Fasting insulin (*μ*U/mL)	14.46 ± 11.94	13.56 ± 11.19	12.02 ± 10.62^a^
Urine microalbumin (mg/L)	87.60 ± 77.89	67.70 ± 64.14^a^	57.17 ± 60.86^a^
C-peptide (ng/mL)	2.62 ± 1.41	2.39 ± 1.06	2.38 ± 1.07
Fibrinogen (g/L)	3.68 ± 1.60	3.34 ± 1.03	3.10 ± 0.79
DR (%)	85.1	74.0	56.1

^a^ *P* < 0.05 versus Q1 and ^b^*P* < 0.05 versus Q2 (one-way ANOVA and post hoc test).

**Table 4:** Odds ratios (95% CI) for risk of DR according to STBC.

	Model 1	*P*	Model 2	*P*	Model 3	*P*
	OR (95% CI)		OR (95% CI)		OR (95% CI)	
Continuous	0.91 (0.89, 0.94)	<0.001	0.93 (0.90, 0.96)	<0.001	0.95 (0.93, 0.99)	0.007
Tertiles						
Q1	1.00 (reference)		1.00 (reference)		1.00 (reference)	
Q2	0.50 (0.32, 0.78)	0.002	0.46 (0.28, 0.73)	0.002	0.51 (0.30, 0.87)	0.014
Q3	0.23 (0.15, 0.35)	<0.001	0.26 (0.16, 0.41)	<0.001	0.37 (0.22, 0.64)	<0.001

Model 1: unadjusted model; Model 2: adjusted for sex, age, BMI, and duration of diabetes; Model 3: adjusted for the variables in model 2 and SBP, DBP, FPG, HbA1c, TC, TG, HDL-C, LDL-C, BUN, Cr, UA, Fasting insulin, urine microalbumin, C-peptide, and fibrinogen.

## Data Availability

The datasets used to support this study are currently under embargo while the research findings are commercialized. Requests for data, 12 months after initial publication, will be considered by the corresponding author.

## References

[1] Animaw W., Seyoum Y. (2017). Increasing prevalence of diabetes mellitus in a developing country and its related factors. *PLoS One*.

[2] Chen L., Magliano D. J., Zimmet P. Z. (2011). The worldwide epidemiology of type 2 diabetes mellitus—present and future perspectives. *Nature Reviews Endocrinology*.

[3] Grubesic T. H., Miller J. A., Murray A. T. (2014). Geospatial and geodemographic insights for diabetes in the United States. *Applied Geography*.

[4] Boyle J. P., Thompson T. J., Gregg E. W., Barker L. E., Williamson D. F. (2010). Projection of the year 2050 burden of diabetes in the US adult population: dynamic modeling of incidence, mortality, and prediabetes prevalence. *Population Health Metrics*.

[5] Whiting D. R., Guariguata L., Weil C., Shaw J. (2011). IDF diabetes atlas: global estimates of the prevalence of diabetes for 2011 and 2030. *Diabetes Research and Clinical Practice*.

[6] Zheng J., Wu Y., Li Z. (2016). Low serum Total bilirubin concentration was associated with increased high sensitive C reactive protein level in patients with impaired glucose tolerance and type 2 diabetes mellitus subjects. *Clinical Laboratory*.

[7] Chen Y. H., Chau L. Y., Chen J. W., Lin S. J. (2008). Serum bilirubin and ferritin levels link heme oxygenase-1 gene promoter polymorphism and susceptibility to coronary artery disease in diabetic patients. *Diabetes Care*.

[8] Soares M. P., Bach F. H. (2009). Heme oxygenase-1: from biology to therapeutic potential. *Trends in Molecular Medicine*.

[9] Cho H. C. (2011). The relationship among homocysteine, bilirubin, and diabetic retinopathy. *Diabetes & Metabolism Journal*.

[10] Sekioka R., Tanaka M., Nishimura T., Itoh H. (2015). Serum total bilirubin concentration is negatively associated with increasing severity of retinopathy in patients with type 2 diabetes mellitus. *Journal of Diabetes and its Complications*.

[11] Huang E. J., Kuo W. W., Chen Y. J. (2006). Homocysteine and other biochemical parameters in type 2 diabetes mellitus with different diabetic duration or diabetic retinopathy. *Clinica Chimica Acta*.

[12] Hughes J. T., Barzi F., Hoy W. E. (2017). Bilirubin concentration is positively associated with haemoglobin concentration and inversely associated with albumin to creatinine ratio among indigenous Australians: eGFR study. *Clinical Biochemistry*.

[13] Shi H. Y., Wang J. R., Cao J., Wang Q. Y., Liu C. P. (2013). Investigation on the difference of intolerance to food between southern and northern middle-aged Chinese and its association with eating habits. *Chinese Journal of Applied Physiology*.

[14] Alberti K. G. M. M., Zimmet P. Z. (1998). Definition, diagnosis and classification of diabetes mellitus and its complications. Part 1: diagnosis and classification of diabetes mellitus. Provisional report of a WHO Consultation. *Diabetic Medicine*.

[15] Zhu B., Wu X., Ning K., Jiang F., Zhang L. (2016). The negative relationship between bilirubin level and diabetic retinopathy: a meta-analysis. *PLoS One*.

[16] Mahabadi A. A., Lehmann N., Möhlenkamp S. (2014). Association of bilirubin with coronary artery calcification and cardiovascular events in the general population without known liver disease: the Heinz Nixdorf Recall study. *Clinical Research in Cardiology*.

[17] Hamamoto S., Kaneto H., Kamei S. (2015). Low bilirubin levels are an independent risk factor for diabetic retinopathy and nephropathy in Japanese patients with type 2 diabetes. *Diabetes & Metabolism*.

[18] Najam S. S., Sun J., Zhang J. (2014). Serum total bilirubin levels and prevalence of diabetic retinopathy in a Chinese population. *Journal of Diabetes*.

[19] Dave A., Kalra P., Gowda B. H., Krishnaswamy M. (2015). Association of bilirubin and malondialdehyde levels with retinopathy in type 2 diabetes mellitus. *Indian Journal of Endocrinology and Metabolism*.

[20] Zhu B., Wu X., Bi Y., Yang Y. (2017). Effect of bilirubin concentration on the risk of diabetic complications: a meta-analysis of epidemiologic studies. *Scientific Reports*.

[21] Coccheri S. (2007). Approaches to prevention of cardiovascular complications and events in diabetes mellitus. *Drugs*.

[22] Vítek L. (2012). The role of bilirubin in diabetes, metabolic syndrome, and cardiovascular diseases. *Frontiers in Pharmacology*.

[23] Temme E. H. M., Zhang J., Schouten E. G., Kesteloot H. (2001). Serum bilirubin and 10-year mortality risk in a Belgian population. *Cancer Causes and Control*.

[24] Fu Y. Y., Kang K. J., Ahn J. M. (2010). Hyperbilirubinemia reduces the streptozotocin-induced pancreatic damage through attenuating the oxidative stress in the Gunn rat. *The Tohoku Journal of Experimental Medicine*.

[25] Abbasi A., Deetman P. E., Corpeleijn E. (2015). Bilirubin as a potential causal factor in type 2 diabetes risk: a Mendelian randomization study. *Diabetes*.

[26] Dudnik L. B., Azyzova O. A., Solovyova N. P., Savchenkova A. P., Pokrovskaya M. A. (2008). Primary biliary cirrhosis and coronary atherosclerosis: protective antioxidant effect of bilirubin. *Bulletin of Experimental Biology and Medicine*.

[27] Hwang H. J., Lee S. W., Kim S. H. (2011). Relationship between bilirubin and C-reactive protein. *Clinical Chemistry and Laboratory Medicine*.

[28] Tapan S., Dogru T., Tasci I., Ercin C. N., Ozgurtas T., Erbil M. K. (2009). Soluble CD40 ligand and soluble P-selectin levels in Gilbert’s syndrome: a link to protection against atherosclerosis?. *Clinical Biochemistry*.

[29] Rocuts F., Zhang X., Yan J. (2010). Bilirubin promotes de novo generation of T regulatory cells. *Cell Transplantation*.

[30] Keyes K., Galea S. (2015). What matters most: quantifying an epidemiology of consequence. *Annals of Epidemiology*.

